# LncRNA miR205HG hinders HNRNPA0 translation: anti‐oncogenic effects in esophageal carcinoma

**DOI:** 10.1002/1878-0261.13142

**Published:** 2021-12-20

**Authors:** Xiaoying Dong, Xuyuan Chen, Di Lu, Dingwei Diao, Xiguang Liu, Shijie Mai, Siyang Feng, Gang Xiong

**Affiliations:** ^1^ Department of Thoracic Surgery Southern Medical University Nanfang Hospital Guangzhou China

**Keywords:** esophageal carcinoma, HNRNPA0, invasion, migration, miR205HG

## Abstract

Esophageal carcinoma (ESCA) affects 4 450 000 people and causes approximately 400 000 deaths annually worldwide, making it the sixth most lethal and eighth most common cancer. Patients with ESCA are often diagnosed at the later stages in which cancer cell metastasis is the main factor contributing to the low 5‐year survival rate (< 20%) of this disease. Long noncoding RNAs (lncRNAs) are a group of regulatory RNAs with a length of > 200 nucleotides but which fail to encode proteins. In this study, by using real‐time quantitative PCR, we found that the expression of the miR205 host gene (miR205HG; a lncRNA) was downregulated in ESCA tumors when compared with normal esophageal tissues or adjacent normal tissues of tumors. Furthermore, we demonstrated that miR205HG modulates the expression of extracellular matrix‐related genes in ESCA cells. In the transwell assay, downregulation of miR205HG contributes to migration and invasion of ESCA cells. In relation to the mechanism, our data show that miR205HG interacts with heterogeneous nuclear ribonucleoprotein A0 (*HNRNPA0*) mRNA and then hamper its translation by interacting with lin‐28 homolog A (LIN28A). Altogether, we highlight that the miR205HG‐HNRNPA0 axis is implicated in the migration and invasion of ESCA cells and that these members of this pathway may serve as therapeutic targets to inhibit metastasis of ESCA.

## Graphical abstract

The lack of early diagnosis for esophageal carcinoma (ESCA) is a main contributor to its low survival rate. In the current study, we found that the downregulation of miR205HG promotes the migration and invasion of ESCA cells by modulating the expression of extracellular matrix genes. Furthermore, we uncovered the mechanism by which miR205HG interacts with LIN28A and then hampers the translation of *HNRNPA0* mRNA. We suggest that miR205HG could potentially be used for the early diagnosis of ESCA and inhibition of metastasis.

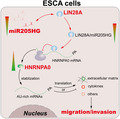

**Abbreviations**
ARE: AU‐rich element
AUC: area under curve of ROC
CLIP: cross‐linking immunoprecipitation
DEGs: differentially expressed genes
EAC: esophageal adenocarcinoma
ECM: extracellular matrix
ESCA: esophageal carcinoma
ESCC: esophageal squamous cell carcinoma
GEO: Gene Expression Omnibus
GEPIA: Gene Expression Profiling Interactive Analysis database
GO analysis: gene ontology enrichment analysis
IB: immunoblotting
lncRNAs: long noncoding RNAs
miR205HG: miR205 host gene
MS: mass spectrometry
MTT: methylthiazolyldiphenyl‐tetrazolium bromide
qPCR: real‐time quantitative PCR
ROC: receiver operator characteristic analysis
shRNA: short hairpin RNA
TCGA: The Cancer Genome Atlas project

## 1 Introduction

Esophageal carcinoma (ESCA) is cancer arising from the esophagus (food pipe that runs between the throat and the stomach), mainly including esophageal squamous cell carcinoma (ESCC) and esophageal adenocarcinoma (EAC) [1, 2, 3]. Every year, ESCA affects 4 450 000 people and causes approximately 400 000 deaths worldwide, making it the sixth most lethal and eighth most common cancer [3, 4, 5]. Generally, patients with ESCA, particularly ESCC, often are diagnosed at the later stages, which likely causes a poor prognosis to patients due to the distant metastasis of cancer cells [6, 7]. Despite advances in diagnosis and treatment in recent years, metastasis still contributes to the main factor causing the low 5‐year survival rate (< 20%) of ESCA [8]. Currently, many genes (such as BRCA1 and SOX4) have been identified to involve in the invasion and metastasis of ESCA, but the effective strategies to control the progression of this disease remain undeveloped [9, 10]. Thus, it is significant to identify key genes implicated in the migration and invasion processes of ESCA.

Long noncoding RNAs (lncRNAs), recently emerged regulatory RNAs in oncology, consist of transcripts with a length of > 200 nucleotides but fail to function as templates for protein synthesis [11, 12]. Accumulating studies by independent groups have shown that a bulk of lncRNAs is aberrantly expressed in tumorigenesis and associated with tumor behavior via mechanisms including miRNA sponging, epigenetic modification, and transcription regulation [12, 13, 14]. Particularly, the expression of lncRNAs such as MALAT1, HOTAIR, AFAP1‐AS1, CASC9, and CCAT1 has been reported to be altered in esophageal cancer, which also contributes to cancer metastasis [15, 16, 17]. MiR205HG (NR_145437, miR205 host gene), a lncRNA also previously named as LINC00510 or NPC‐A‐5, is a lncRNA with a length of 895 nucleotides transcribed from a 3.7 kb genomic sequences within 1q32.2 band of human [18]. In the tumorigenesis of cervical carcinoma, miR205HG was found to be aberrantly expressed and play fundamental roles by modulating KRT17 expression [19]. More recently, Liu *et al*. [20] reported that miR205HG expression in lung squamous cell carcinoma tissues and cell lines was upregulated, which also expedites cell proliferation and cancer progression. These reports reveal the versatility of miR205HG in tumorigenesis. More interestingly, after re‐analyzing the miR205HG expression pattern in ESCA tissues using the published datasheet in the Gene Expression Omnibus (GEO) database, we found that miR205HG is substantially downregulation in tumors compared with the adjacent normal tissues. This discovery impelled us to explore the role of miR205HG in the pathogenesis of ESCA.

Briefly, we first reported the expression pattern of miR205HG in ESCA tissues was downregulated when compared with normal esophageal tissues or adjacent normal tissues of tumors. Furthermore, we demonstrated miR205HG modulates the expression of extracellular matrix (ECM)‐related genes in ESCA cells, and downregulation of miR205HG contributes to migration and invasion of ESCA cells. In mechanism, our data show that miR205HG interacts with *HNRNPA0* mRNA and then hamper its translation. Altogether, we highlight that miR205HG/HNRNPA0 axis is implicated in the migration and invasion of ESCA cells, which may serve as therapeutic targets to inhibit ESCA metastasis.

## 2 Materials and methods

### 2.1 Patients

A total of 153 esophageal carcinoma patients (including 127 ESCC, 11 EAC, and 15 others) with histological validation were enrolled in the hospital Southern Medical University Nanfang Hospital from February 2014 to October 2019. The tumor and adjacent normal specimens were collected and immediately immersed in RNAlater solution from Thermo Fisher Scientific (Cat: AM7021, Waltham, MA, USA). All the specimens were stored at −80 °C for a long‐time. Three independent oncologists checked the pathological sections and classified the pathological stage for each patient using the 7th edition of the Union for International Cancer Control‐American Joint Committee on Cancer (UICC‐AJCC) tumor, node, metastasis (TNM) staging system. The related clinical features of patients are presented in Table 1. Notably, the protocols and procedures used in this study were approved by the Ethics Committee of the Southern Medical University Nanfang Hospital. All participates (or their relatives) signed the written informed consent. The study methodologies conformed to the standards set by the Declaration of Helsinki.

**Table 1:** Clinical futures of patients with ESCA in this study.

Clinical variables	
Age (years) (mean, min–max)	64.5 (54–76)
Sex	
Male	133 (86.9%)
Female	20 (13.1%)
Body mass index	22.3 ± 2.2
Performance	
0	135 (88.2%)
I–IV	18 (11.7%)
Tobacco	
No	13 (8.4%)
Yes	140 (91.5%)
Alcohol	
No	21 (13.7%)
Yes	132 (86.2%)
Histological type	
Squamous cell carcinoma	127 (83.0%)
Adenocarcinoma	11 (7.2%)
Others	15 (9.8%)
Pathological stage	
I	57 (37.3%)
II	52 (33.9%)
III	44 (28.8%)
Tumor infiltrating lymphocytes	
No	48 (31.3%)
Yes	105 (68.6%)
Total	153

### 2.2 RNA isolation and real‐time quantitative PCR (qPCR)

Total RNA of tissues and cell lines was isolated using QIAsymphony RNA Kit (Cat: 931236; QIAGEN, Germantown, MD, USA) according to the manufacturer's instructions. Next, reverse transcription was conducted using a PrimeScript kit (Takara, Dalian, China) by adding 5 μg total RNA. Finally, the relative quantification of RNA was then performed on CFX96 Touch™ Real‐Time PCR Detection System (Bio‐Rad, Hercules, CA, USA) using TB Green™ Fast qPCR Mix (Takara) following the provided manual. The 2^ΔΔCT^ method was used for the relative quantitation of RNA gene expression in which GAPDH serves as internal controls. All used oligos are presented in Table 2.

**Table 2:** Oligos used in this study for real‐time quantitative PCR.

Targets	Direction	Sequences (5′ → 3′)
BGN	Forward	GAGACCCTGAATGAACTCCACC
	Reverse	CTCCCGTTCTCGATCATCCTG
COL10A1	Forward	GGGGCTAAGGGTGAAAGGG
	Reverse	GGTCCTCCAACTCCAGGATCA
COL5A1	Forward	TACAACGAGCAGGGTATCCAG
	Reverse	ACTTGCCATCTGACAGGTTGA
ICAM1	Forward	ATGCCCAGACATCTGTGTCC
	Reverse	GGGGTCTCTATGCCCAACAA
LAMC2	Forward	TGGAGAACGCTGTGATAGGTG
	Reverse	CAGGAGACCCATTTCGTTGGA
MMP1	Forward	GGGGCTTTGATGTACCCTAGC
	Reverse	TGTCACACGCTTTTGGGGTTT
MMP3	Forward	CGGTTCCGCCTGTCTCAAG
	Reverse	CGCCAAAAGTGCCTGTCTT
MYH11	Forward	GGTCACGGTTGGGAAAGATGA
	Reverse	GGGCAGGTGTTTATAGGGGTT
SPARC	Forward	CCCATTGGCGAGTTTGAGAAG
	Reverse	CAAGGCCCGATGTAGTCCA
SPINK5	Forward	TGCTTTTCGGCCCTTTGTTAG
	Reverse	CACACATTGCACACTTATTGCC
FAM3D	Forward	CTGCCCAGCCAACTACTTTG
	Reverse	CTCCCGTGGTTCCATTCAC
FAM3B	Forward	ACACCTATGCCTACAGGTTACT
	Reverse	CAAAACATCGTGTTGCTGTCAC
IL1B	Forward	AGCTACGAATCTCCGACCAC
	Reverse	CGTTATCCCATGTGTCGAAGAA
IL1RN	Forward	CATTGAGCCTCATGCTCTGTT
	Reverse	CGCTGTCTGAGCGGATGAA
IL32	Forward	TGGCGGCTTATTATGAGGAGC
	Reverse	CTCGGCACCGTAATCCATCTC
SLURP1	Forward	GGCCCTCAAGTGCTACACC
	Reverse	GTTGAAGGGGTACTCTGCCT
SPP1	Forward	CTCCATTGACTCGAACGACTC
	Reverse	CAGGTCTGCGAAACTTCTTAGAT
HNRNPA0	Forward	TGGCTTCGTGACCTACTCCAA
	Reverse	GGCCTCCGACAAAGAGCTT
GAPDH	Forward	GGAGCGAGATCCCTCCAAAAT
	Reverse	GGCTGTTGTCATACTTCTCATGG
miR205HG	Forward	ATCTCTCAAGTACCCATCTTGGA
	Reverse	GGCCTCATGGTTGTCAGCTC

### 2.3 Cell culture

TE4 (Cat: RCB2097) and TE6 (RCB1950) cells were purchased from the cell bank of RIKEN BioResource Research Center (Ibaraki, Japan) and cultured in RPMI‐1640 plus 10% FBS (Gibco, Rockville, MD, USA) and 50 μg·mL^−1^ penicillin/streptomycin (P/S; Gibco). OE19 (Cat: 96071721), FLO‐1 (Cat: 11012001), SK‐GT‐4 (Cat: 11012007), and OE21 (Cat: 96062201) cells were purchased from the European Collection of Authenticated Cell Cultures (ECACC) and cultured in RPMI‐1640 (or DMEM in the case of FLO‐1) plus 2 mm Glutamine, 10% FBS, and 50 μg·mL^−1^ P/S. All the cells were maintained in a CO_2_ incubator with a condition of 5% CO_2_ and 37 °C.

### 2.4 Immunoblotting

For the preparation of immunoblotting (IB) samples, the protein of cell lysates was quantified using Easy II Protein Quantitative Kit (based on BCA; TransGen Biotech, Beijing, China). Next, the equal quantification of protein samples was electrophoresed using SDS/PAGE gel (containing 1% SDS, 1.5 m Tris pH8.8) and was transferred to nitrocellulose (NC) membrane. After blocking with 5% (w/v) skim milk at room temperature for 1 h, the membranes were incubated with primary antibodies in recommended dilution at 4 °C overnight. The next day, after washing with 1×TBST three times (each for 5 min), the membranes were incubated with HRP‐conjugated secondary antibodies (with a dilution of ~ 1 : 2000–5000) at room temperature for 1 h. After washing with 1×TBST for 3 × 5 min, the membranes were detected using Tanon™ High‐sig ECL Western Blotting Substrate Kit (Cat: 180‐501, Tanon, Shanghai, China). The protein bands were scanned and analyzed using Bio‐Rad ChemiDoc MP. Primary antibodies of HNRNPA0 (Cat: #5545), LIN28A (Cat: #8706), FLAG (Cat: #14793), COL5A1 (Cat: #86903), MMP1 (Cat: #54376), and GAPDH (Cat: #5174) were purchased from Cell Signaling Technology (Danvers, MA, USA). SPINK5 (Cat: AF8515) was purchased from R&D System (Minneapolis, MN, USA).

### 2.5 Bioinformatics analysis

To explore the functional genes in the metastasis of ESCA, we re‐analyzed the published transcriptomic data of esophageal squamous carcinoma (ESCC) and adjacent nontumor tissues from the GEO (GSE149609). In this datasheet, 10 paired samples of tumor and adjacent nontumor were included. R language packages of GEOquery, Limma, ggplot2, and ClusterProfiler were used for data analysis and visualization in which *P*‐value < 0.01 and fold change > 2 were selected as the cutoff.

Besides, the online tool of the Gene Expression Profiling Interactive Analysis (GEPIA, http://gepia2.cancer‐pku.cn/) was used for miR205HG expression level in ESCA patients (*n* = 286) from The Cancer Genome Atlas (TCGA) and normal (*n* = 182) esophageal tissues from the Genotype‐Tissue Expression (GTEx) database. The cutoff of adjusted *P*‐value < 0.01 and log2(fold change) > 2 was used for the identification of differentially expressed genes (DEGs) in GEPIA.

The targets of lncRNA were predicted using the online tools of lnctar (http://www.cuilab.cn/lnctar) and ENCOR (http://starbase.sysu.edu.cn/). The candidates were selected based on: (a) low sequences binding free energy and (b) functional potential.

### 2.6 RNA pull‐down assay

Biotin‐labeled miR205HG and GFP (as a negative control) were chemically synthesized by Sangon Biotech (Shanghai, China). The dry powder of RNA was resolved into DEPC water containing RNase‐free DNase I (Cat: EN0521; Thermo Fisher). RNA solution was heated at 95 °C for 5 min and then cooled on ice for 5 min to recover RNA secondary structure, followed by reacting with streptavidin agarose beads (Invitrogen, Carlsbad, CA, USA) for 12 h. For the RNA pull‐down assay, cells (~ 0.5 × 10^7^) were collected using mild RIPA Lysis Buffer (Cat: PH0317, Wuhan, China), and the obtained lysates were incubated with RNA captured beads at 4 °C. After 2 h, the beads were briefly washed three times using 1× wishing buffer containing 50 mm Tris/HCl, 150 mm NaCl, 1 mm MgCl_2_, and 0.05% NP‐40. After the final washing, the supernatant of each tube was drained using a vacuum pump, and the obtained beads were resuspended in 1× SDS loading buffer and boiled at a metal block for 10 min. The samples were subjected to immunoblotting analysis.

### 2.7 Cross‐linking immunoprecipitation of RNA protein

To estimate physical interaction between LIN28A protein and miR205HG, a cross‐linking immunoprecipitation (CLIP) assay was conducted as previously reported protocol [21]. Briefly, TE6 and OE21 cells (1 × 10^7^) were washed using 1× PBS and UV cross‐linked at a dose of 400 mJ·cm^−2^. Then, cells were lysed using mild RIPA Lysis Buffer (Cat: PH0317, Wuhan, China) containing protease inhibitor (Cat. HY‐K0010; MedChemExpress, Shanghai, China) and RNase inhibitor (Cat: AM2694; Thermo Fisher). After precleaning with protein G sepharose beads (Cat: P3296; Sigma, Darmstadt, Germany), cell lysates were incubated with LIN28A antibody (1 μg) at 4 °C for 3 h. Next, the solution containing antibody–RNA complexes was incubated with BAS‐blocked Protein G sepharose beads at 4 °C for 3 h. After being washed with washing buffer containing protease and RNase inhibitor, the beads bound with RNP complex were eluted for the subsequent RNA isolation.

### 2.8 MTT assay

To determine the proliferation ability of cells, TE4, TE6, OE19, and OE21 cells (10^4^ cells/well) were seeded into a 96‐well in three successive days with an interval of 0.5 days. Twenty‐four hours after the last seeding, cells were treated with 20 μL MTT solution (Methylthiazolyldiphenyl‐tetrazolium bromide, Cat: HY‐15924; MedChemExpress) for 4 h at 37 °C. Subsequently, 150 μL DMSO was added into the culture medium to dissolve the obtained violet crystal. After incubation at room temperature for 15 min, cells were subjected to proliferation curve determination using a SpectraMax iD analyzer (Molecular Devices, San Jose, CA, USA).

### 2.9 Wound‐healing assay

To estimate the migrative ability of cells, TE4/TE6/OE19/OE21 cell lines (2.5 × 10^5^ cells/well) were seeded into a 12‐well plate for wound‐healing assay. Twenty‐four hours later, the confluent cell monolayer was wounded using a 10‐μL sterile pipette tip. After healing for 1, 2, and 3 days, the wound width was determined using an Olympus CKX53‐inverted microscope (Itasca, IL, USA).

### 2.10 Transwell assay

TE6 and OE21 cell lines (3 × 10^4^ cells/well) were seeded on the upper side of the Matrigel‐coated transwell chamber. The medium of the transwell chamber upper side was supplied with 10% FBS, whereas the medium of the bottom side was supplied with 20% FBS. The cells of the bottom side were allowed to grow for 24 h and then were fixed using the 4% paraformaldehyde for 30 min. After being stained using crystal violet for 30 min, the cells migrated to the bottom side were imaged using a microscope for further cell quantification.

### 2.11 Statistical analysis

The data in the present study were expressed as mean ± SD of which the duplication was indicated in the figure legend. The Student's *t*‐test was used for the comparison between two groups; one‐way ANOVA was used for comparison with more than two groups. The software of graphpad prism 7 (San Diego, CA, USA) was used for statistical analysis. A *P*‐value < 0.05 was considered as statistical significance.

## 3 Results

### 3.1 MiR205HG is downregulated in esophageal squamous cell carcinoma

**Fig. 1:**
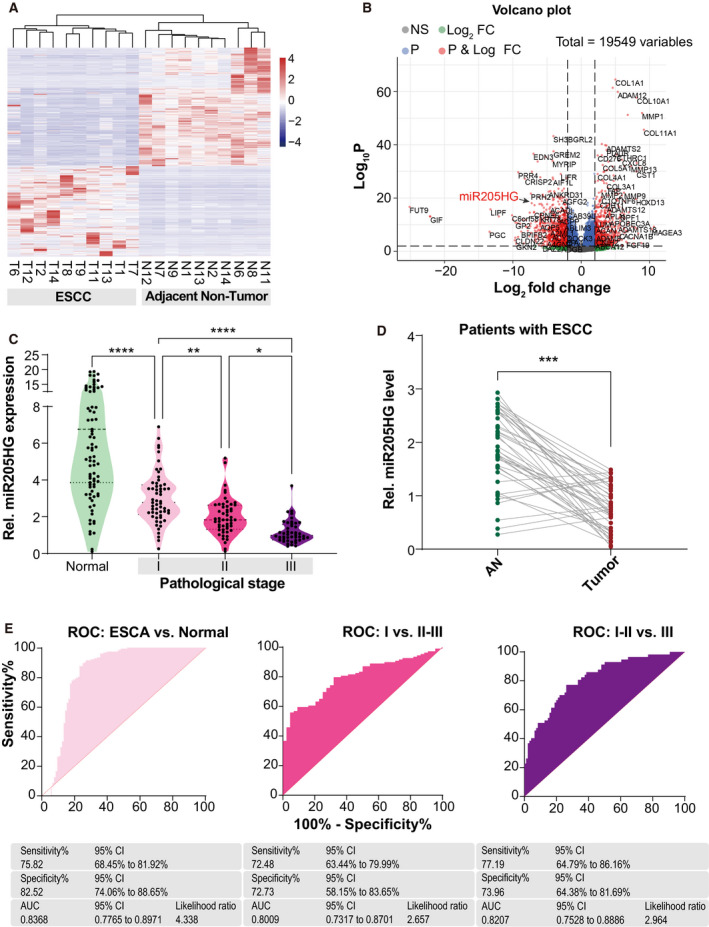
MiR205HG is downregulated in ESCA. (A, B) Heatmap (A) and Volcano plot (B) of the DEGs between ESCC tumor and their adjacent nontumor tissues. GSE149609 RNAseq datasheet was used for differential expression analysis. T, tumor; N, adjacent nontumor; FC, fold change; NS, no significance. FC > 2 and *P*‐value < 0.01 serve as the cutoff. (C) MiR205HG expression in normal esophageal tissues and ESCA tumors was determined by real‐time qPCR. One hundred and fifty‐three patients with ESCA and 94 normal donors were enrolled. ESCA, esophageal carcinoma; pathological stage I/II/II, TNM stage I/II/III; *P < 0.05, **P < 0.01, and ****P < 0.0001 by Student's *t*‐test. (D) MiR205HG expression in ESCC tumors and their paired nontumor tissues were determined by real‐time qPCR. Forty‐two patients with ESCC were enrolled. AN, adjacent nontumor; ***P < 0.001 by paired Student's *t*‐test. (E) ROC analysis for ESCA diagnosis using miR205HG expression level. AUC, area under curve of ROC; 95% CI, 95% confidence interval.

To investigate the pathogenesis of ESCC, we re‐analyzed a recently published RNAseq datasheet (GSE149609, with 10 tumors and 10 adjacent nontumor tissues) from the GEO database because this datasheet has not been fully explored. Under the cutoff of *P*‐value < 0.01 and fold change > 2, we obtained 1181 DEGs between tumor and adjacent nontumor groups from a total of 19 549 genes (Fig. 1A,B). Among these DEGs, we were fascinated by a lncRNA, miR205HG (tumor vs. adjacent nontumor, log2(fold change) = −2.123, *P*‐value = 5.03e‐15), because its expression aberration has been frequently observed in many other cancers including lung squamous cell carcinoma and cervical cancer [3, 20, 22]. In another way, expression data from Genotype‐Tissue Expression Project (GTEx) revealed that miR205HG is highly expressed in esophagus mucosa (Fig. S1A). Consistently, we also found that miR205HG was dramatically downregulated in the ESCA samples from the Cancer Genome Atlas (TCGA) database when compared with normal esophageal tissues from the GTEx database (Fig. S1B). Therefore, we determined the expression level of miR205HG in 153 ESCA (including 57 TNM stage I, 52 TNM stage II, and 44 TNM stage III) tumors and 94 normal esophageal tissues. Our results showed that the expression level of miR205HG in ESCA tissues was significantly downregulated compared with that in normal esophageal tissues, and negatively correlated with tumor stages (Fig. 1C). Meanwhile, we found that, in 42 ESCC patients, the expression level of miR205HG in tumors was significantly lower than that of their adjacent nontumor tissues (Fig. 1D). However, miR205HG expression in EAC tissues was close to that of adjacent nontumor tissues (Fig. S1C). Besides, we performed the receiver operator characteristic (ROC) analysis for the ESCA diagnosis using miR205HG expression level. Our data showed that miR205HG distinguished ESCA from normal esophageal tissues with a sensitivity of 75.8% and a specificity of 82.5% (AUC, 0.83; 95% CI, [0.77–0.89]; likelihood ratio, 4.3) (Fig. 1E). MiR205HG also efficiently recognized stage I ESCA from stage II–III ones (AUC, 0.80; 95% CI, [0.73–0.87]; likelihood ratio, 2.6) and stage III ESCA from stage I–II ones (AUC, 0.82; 95% CI, [0.75–0.88]; likelihood ratio, 2.9). Collectively, our evidence shows that miR205HG expression level is reduced in ESCA tumors, and low miR205HG expression is unfavorable for ESCA patients.

### 3.2 MiR205HG modulates ECM‐related genes expression

**Fig. 2:**
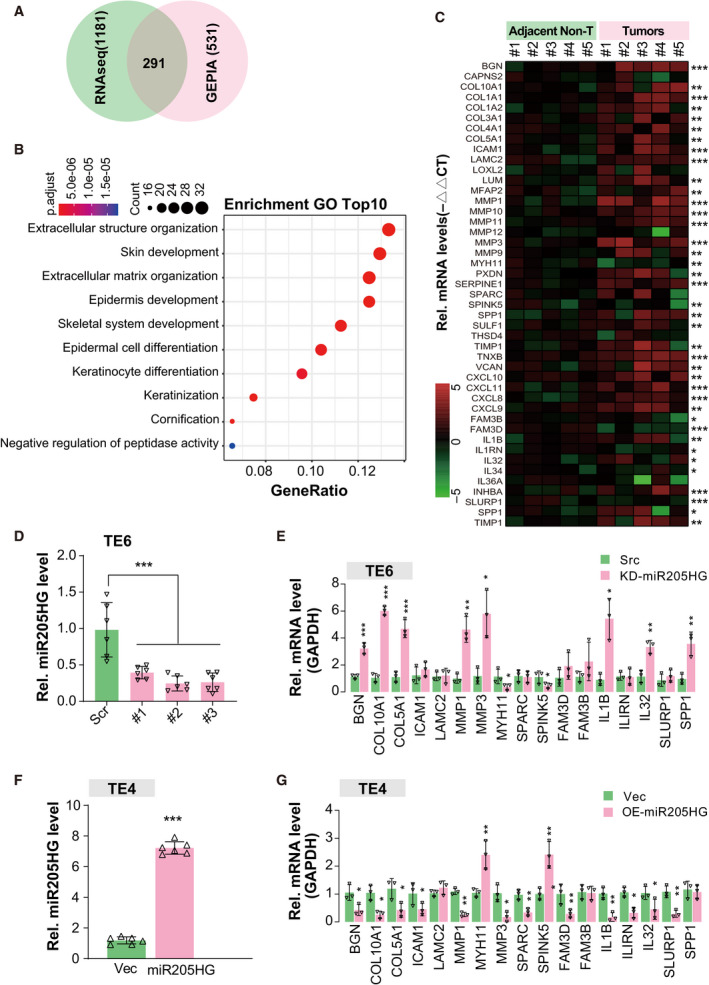
MiR205HG modulates ECM‐related genes expression. (A) Venn graph shows the overlap between the DEGs of GSE149609 and ESCA from TCGA. One thousand one hundred and eighty‐one DEG of GSE149609 are the same as Fig. 1A,B. Five hundred and thirty‐one DEGs of ESCA from The Cancer Genome Atlas project (TCGA) were obtained by the Gene Expression Profiling Interactive Analysis (GEPIA) tool with the cutoff of fold change > 2 and *P*‐value < 0.01, compared with normal esophageal tissues. (B) Gene ontology enrichment analysis (cell component) of overlap DEGs in A. (C) Heatmap shows the real‐time qPCR results of the indicated genes. Five paired samples of ESCA tumor and adjacent nontumor were used for relative quantification. *P < 0.05, **P < 0.01, and ***P < 0.001 by paired Student's *t*‐test. (D) MiR205HG expression levels in TE6 cells were determined by real‐time qPCR. Three independent targets for miR205HG were used, indicated by #1/2/3. Scr, scramble shRNA; KD‐miR205HG, knockdown of miR205HG; *N* = 6 (mean ± SD), ***P < 0.001 by one‐way ANOVA. (E) Real‐time qPCR determined the expression level of indicated genes. Three miR205‐knockdown TE6 cell lines (as in D) were pooled together. Src, scramble control; *N* = 3 (mean ± SD); *P < 0.05, **P < 0.01, and ***P < 0.001 by Student's *t*‐test. (F) MiR205HG expression levels in TE4 cells were determined by real‐time qPCR. Vec, vector control; OE‐miR205HG, overexpression of miR205HG; *N* = 6 (mean ± SD), ***P < 0.001 by Student's *t*‐test. (G) Real‐time qPCR determined the expression level of indicated genes. TE4 cell lines in F were used. *N* = 3 (mean ± SD); *P < 0.05 and **P < 0.01 by Student's *t*‐test.

Meanwhile, we conducted a differential expression analysis of ESCA RNAseq data from TCGA using the tool of GEPIA. The results showed that 531 genes were aberrantly expressed of which 291 ones overlap with the DEGs in GSE149609 (Fig. 2A). After gene ontology enrichment (GO) analysis using the overlap DEG sets, we found that these genes were enriched in ECM‐related components (Fig. S1D and Fig. 2B). Intriguingly, these observations were almost further validated by our real‐time qPCR results of five pairs of tumor and adjacent nontumor samples, such as COL1A1, MMP1, MMP10, CXCL8, and IL1B (Fig. 2C). Therefore, we wondered whether miR205HG executes a remarkable effect on the expression of ECM‐related components and cytokines as mentioned above. We knocked down miR205HG expression using short hairpin RNA (shRNA) vector (with three independent targets) in TE6 and OE21, two miR205HG‐high expressed ESCC cell lines (Fig. S2A). Interestingly, we found that knockdown of miR205HG expression in TE6 cells promoted BGN, COL10A1, COL5A1, MMP1, MMP3, IL1B, IL32, and SPP1 expression but restrained SPINK5 expression (Fig. 2D,E). Likewise, similar results were observed in miR205HG‐knockdown OE21 cells (Fig. S2A,B). In contrast, our data of TE4 cells (a miR205HG‐low expressed ESCC cell lines) with miR205HG overexpression revealed that the ectopic miR205HG enhances MYH11 and SPINK5 expression but suppressed BGN, COL10A1, COL5A1, MMP1, MMP3, and IL1B expression (Fig. 2F,G). Meanwhile, we manipulated the expression level of miR205HG in three EAC cell lines including FLO‐1, OE19, and SK‐GT‐4, and then detected the ECM‐related genes expression (Fig. S2C–E). Similar to ESCC cells, we also found that overexpression of miR205HG inhibited but knockdown promoted the ECM‐related genes expression (Fig. S2C–E). Consistently, our immunoblotting results also confirmed the protein level alterations of targets mentioned above including COL5A1, MMP1, and SPINK5 in TE6 and OE19 cells (Fig. S2F). Altogether, we demonstrated that miR205HG modulates ECM‐related gene expression both in ESCC and EAC *in vitro*.

### 3.3 MiR205HG influences proliferation, migration, and invasion of ESCA cells

**Fig. 3:**
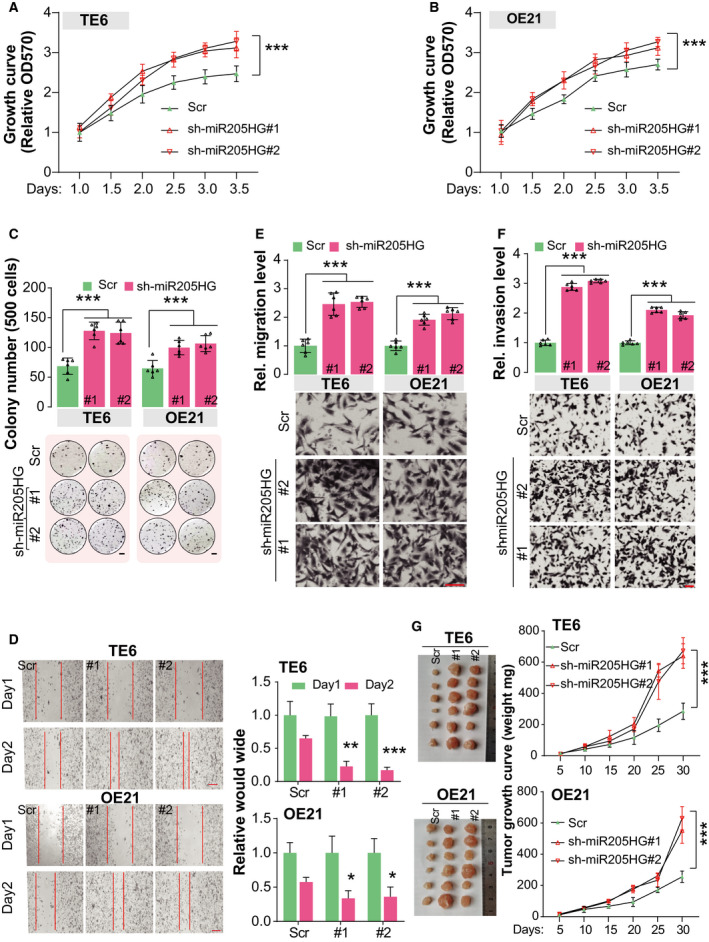
MiR205HG influences proliferation, migration, and invasion of ESCA cells. (A, B) Cell proliferation of TE6 (A) and OE21 (B) cells was determined by MTT assay. MiR205HG was stably knocked down using shRNA vectors. # indicates independent targets. Scr, scramble control; *N* = 4 (mean ± SD), ***P < 0.001 by two‐way ANOVA. (C) Colony formation assay of TE6 and OE21 cells. Cells in A and B were used. Scale bar, 2 mm; *N* = 4 (mean ± SD), ***P < 0.001 by one‐way ANOVA. (D) Migration ability of TE6 and OE21 cells was estimated using a wound‐healing assay. Cells in A and B were used. Scale bar, 200 µm; *N* = 6 (mean ± SD); *P < 0.05, **P < 0.01 and ***P < 0.001 by paired Student's *t*‐test. (E, F) Cell migration (E) and invasion (F) were determined by transwell assay. Cells in A and B were used. In the transwell assay of invasion, the chambers were coated using Matrigel. Scale bar, 100 µm; *N* = 6 (mean ± SD), ***P < 0.001 by one‐way ANOVA. (G) Tumorigenicity of TE6 and OE21 cells was estimated using xenograft assay. Cells in A were used. Scale bar, 1 cm; *N* = 4 (mean ± SD), ***P < 0.001 by two‐way ANOVA.

The ECM, as a physical scaffold and signaling platform of cells in tissues, has been frequently observed to be perturbed in various tumors [23, 24, 25]. Tumor ECM also has been demonstrated to promote the growth, survival, and invasion of cancer cells to drive metastasis in most cancer patients [26, 27, 28, 29]. Combined with the findings mentioned above, we hypothesized that miR205HG may play a role in the tumorigenicity of ESCA cells. Firstly, we knocked down miR205HG expression in TE6 and OE21 cells using a shRNA vector and overexpressed it in TE4 and OE19 cells (Fig. S3B). MTT assay results showed that knockdown of miR205HG in TE6 and OE21 cells significantly enhanced cell proliferation (Fig. 3A,B). Meanwhile, cell proliferation of TE4 and OE19 cells was substantially restrained after miR205HG overexpression (Fig. S3C,D). In colony formation assay, we found that knockdown of miR205HG in TE6 and OE21 cells promoted, but overexpression in TE4 and OE19 cells inhibited colony formation (Fig. 3C and Fig. S3E). In another way, we also carried out the would‐healing and transwell assay to estimate the migration and invasion of ESCA cell lines after manipulating miR205HG expression. The would‐healing assay revealed that cell migration was enhanced after the knockdown of miR205HG in TE and OE21 cells (Fig. 3D). Consistently, transwell assay results showed that knockdown of miR205HG promoted cell migration, supported by the significantly increased migration cell number (more than 2‐fold of scramble groups) in miR205HG‐knockdown TE6 and OE21 cells (Fig. 3E). Similarly, after the knockdown of miR205HG expression, the invasiveness of TE6 and OE21 cells also was enhanced by 2‐ to 3‐fold (Fig. 3F). Furthermore, we found that overexpression of miR205HG in TE4 and OE19 cells restrained the cell migration and invasion in the transwell assay (Fig. S3F,G). Besides, we explored miR205HG's role in tumorigenesis of ESCA cells *in vivo* using xenograft assay. Our findings exhibited that knockdown of miR205HG in TE6 and OE21 cells significantly promoted tumor growth in nude mice (Fig. 3G). Taken together, our findings indicated that miR205HG influences proliferation, migration, and invasion of ESCA cells.

### 3.4 MiR205HG restrains translation of HNRNPA0 by directly interacting with its mRNA

**Fig. 4:**
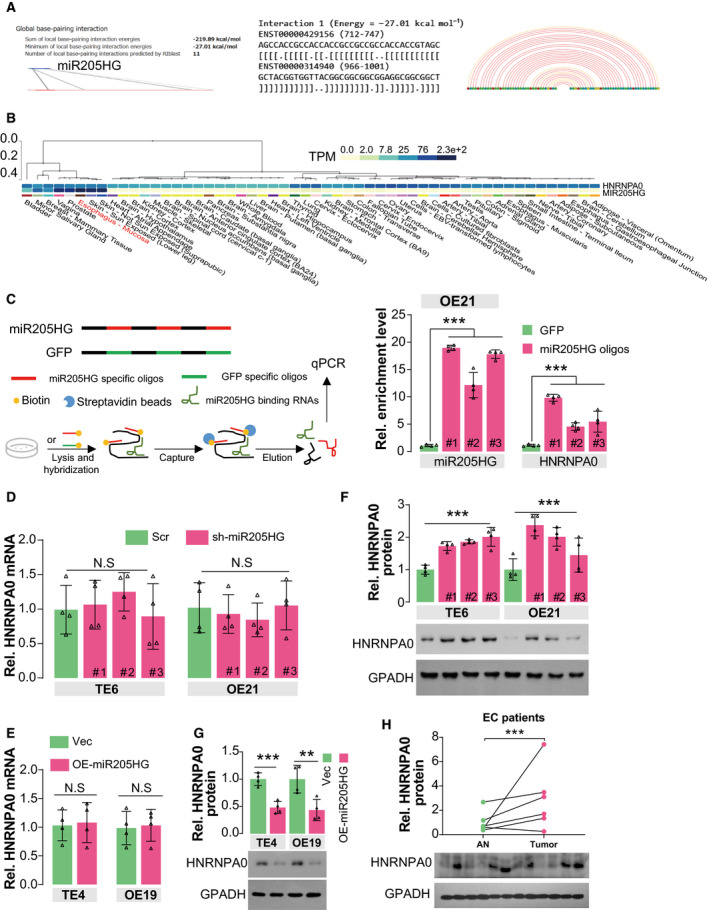
MiR205HG restrains translation of HNRNPA0 by directly interacting with its mRNA. (A) The interaction between miR205HG and *HNRNPA0* mRNA was predicted by the LncTar algorithm. (B) Expression levels of miR205HG and *HNRNPA0* mRNA in human tissues. Expression data derived from the GTEx database. TPM, Transcripts Per Million in reads in RNAseq. (C) RNA pull‐down assay showed the interaction between miR205HG and HNRNPA0. 10^7^ OE21 cells were lysed and hybridized using biotin‐labeled miR205HG specific oligos. The lysates were incubated with Streptavidin beads. Finally, the captured RNA complex was eluted and quantified by real‐time qPCR. #1/2/3, different miR205HG specific oligos. *N* = 3 (mean ± SD), ***P < 0.001 by one‐way ANOVA. (D, E) *HNRNPA0* mRNA level was determined by real‐time qPCR. Cells in Fig. 2D/F and Fig. S2B/D were used in the experiments. Scr, scramble shRNA; sh‐miR205HG, shRNA targeting miR205HG; Vec, vector; OE‐miR205HG, overexpression of miR205HG; *N* = 4 (mean ± SD); N.S, no significance by one‐way ANOVA (D) and Student's *t*‐test (E). (F–H) The protein level of HNRNPA0 was determined by IB. The cells in D and E were used. *N* = 4 (mean ± SD), ***P < 0.001 by one‐way ANOVA (F), **P < 0.01 and ***P < 0.001 by Student's *t*‐test (G); *N* = 3 (mean ± SD), ***P < 0.001 by paired *t*‐test (H).

To investigate the mechanisms that miR205HG achieves its effects on the migration and invasion of ESCA cells, we predicted its putative targets using two different bioinformatic tools including ENCOR and LncTar. Interestingly, our attention was attracted by the gene of HNRNPA0 (heterogeneous nuclear ribonucleoprotein A0), because (a) the RNA interaction between miR205HG and *HNRNPA0* has a relatively low binding free energy (sum of local base‐pairing interaction energies: −219.89 kcal·mol^−1^, and a minimum of local base‐pairing energies: −27.01 kcal·mol^−1^, Fig. 4A), and (b) HNRNPA0 functionally binds AU‐rich element (ARE)‐containing mRNAs to simultaneously dozens of mRNAs stability [30]. Moreover, we found that, referring to the expression data from GTEx, *HNRNPA0,* and miR205HG are quite highly expressed in esophagus tissues (Fig. 4B). Therefore, we suspected that the regulatory effect of miR205HG on ECM‐related gene expression may be mediated by HNRNPA0. In RNA pull‐down assay, we observed that miR205HG specific oligos (3 independent targets) effectively pulled down *HNRNPA0* transcripts (Fig. 4C), indicating there is an interaction between miR205HG and *HNRNPA0*. However, neither knockdown of miR205HG in TE6 and OE21 cells nor overexpression of miR205HG in TE4 and OE19 cells influenced the mRNA level of *HNRNPA0* (Fig. 4D,E). Surprisingly, we found the protein level of HNRNPA0 in TE6 and OE21 cells was up‐regulated by miR205HG knockdown (Fig. 4F), while ectopic miR205HG also decreased HNRNPA0 protein level in TE4 and OE19 cells (Fig. 4G). Also, we observed that the protein level of HNRNPA0 in ESCA tumors was considerably higher than that in adjacent normal tissues (Fig. 4H). These data collectively reveal that miR205HG modulates the translation efficiency of HNRNPA0 but not its transcription.

### 3.5 MiR205HG suppresses HNRNPA0 translation by interacting with LIN28A

**Fig. 5:**
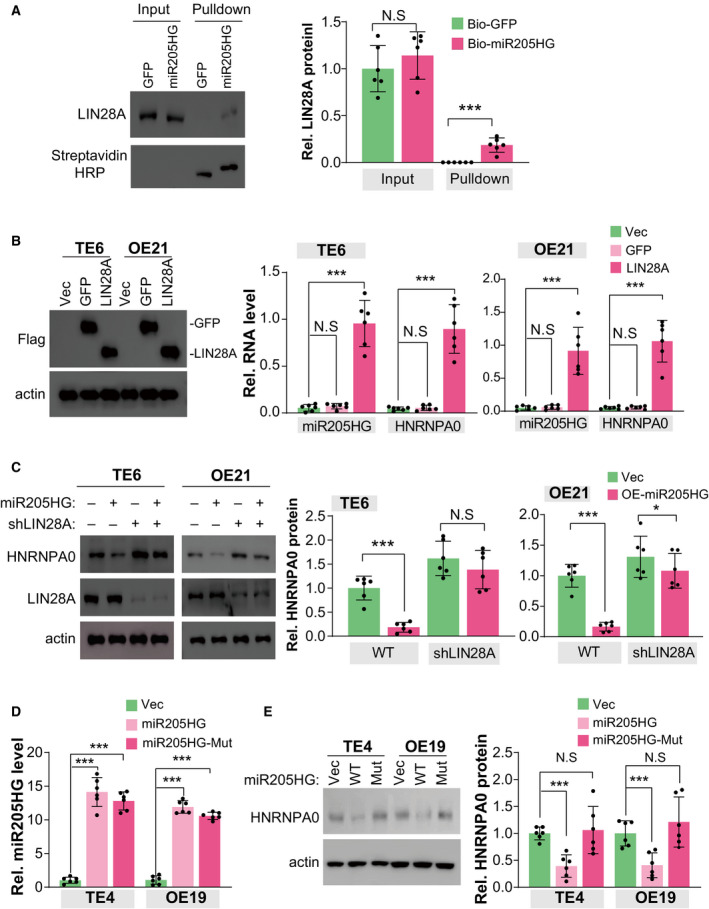
MiR205HG suppresses HNRNPA0 translation by interacting with LIN28A. (A) The interaction between LIN28A protein and miR205HG was determined by RNA pull‐down assay. Streptavidin agarose beads bound with biotin‐labeled miR205HG (or biotin‐labeled GFP) were incubated with TE6 cell lysate (˜ 0.5 × 10^7^). The protein level of miR205HG binding LIN28A was determined by IB. *N* = 6 (mean ± SD); N.S., no significance and ***P < 0.001 by student's *t*‐test. (B) The interaction between LIN28A protein and miR205HG/HNRNPA0 RNA was determined by CLIP assay. 1 × 10^7^ TE6 and OE21 cells overexpressed with Flag‐tag LIN28A or GFP (as negative control) were used. The miR205HG and HNRNPA0 RNA level bound by LIN28A was determined by RT qPCR. *N* = 6 (mean ± SD); N.S., no significance and ***P < 0.001 by Student's *t*‐test. (C) The protein level of HNRNPA0 and LIN28A was determined by IB. The TE6 and OE21 cell lines as indicated were used. *N* = 3 (mean ± SD); N.S., no significance, *P < 0.05 and ***P < 0.001 by Student's *t*‐test. (D) MiR205HG level was determined by real‐time qPCR. TE4 and OE19 overexpressed with wild‐type (WT) miR205HG and ‘GGAG’‐mutant miR205HG (Mut) were used. *N* = 6 (mean ± SD), ***P < 0.001 by Student's *t*‐test. (E) The protein level of HNRNPA0 was determined by IB. The TE4 and OE19 cell lines as D were used. *N* = 3 (mean ± SD); N.S, no significance and ***P < 0.001 by Student's *t*‐test.

To investigate the exact mechanism for miR205HG regulating the translation of *HNRNPA0* mRNA, we carried out a biotin‐labeled miR205HG RNA pull‐down assay, followed by mass spectrometry (MS) to identify its binding proteins. Fortunately, we observed several RNA binding proteins in the MS results (biotin‐labeled GFP as negative control), such as TAF15, LIN28A, FUSIP1, HNRNPH2, and GSTO1 (Table 3). Intriguingly, we validated the interaction between miR205HG and LIN28A using immunoblotting (Fig. 5A). Meanwhile, we found that ectopic LIN28A protein in TE6 and OE21 cells specifically interacted with miR205HG and *HNRNPA0* mRNA (Fig. 5B). In another line, our evidence showed that, in miR205HG‐knockdown TE6 and OE21 cells, the interaction between *HNRNPA0* mRNA and LIN28A protein was weakened (Fig. S4A), indicating that miR205HG mediates the interaction. Moreover, we observed that knockdown of LIN28A using shRNA in TE6 and OE21 cells largely restrained the effect of ectopic miR205HG on HNRNP0 protein level (Fig. 5C), suggesting that LIN28A participates in the regulatory mechanism of miR205HG to HNRNPA0 protein. Previously, Cho *et al*. [31] reported that LIN28A is a suppressor of endoplasmic reticulum‐associated translation in embryonic stem cells by binding ‘GGAG’ sequences in the terminal loop of let‐7 precursors. We also observed a ‘GGAG’ motif in the base‐paired region of miR205HG and *HNRNPA0* mRNA, and therefore, we constructed a ‘GGAG’‐mutant miR205HG plasmid (miR205HG‐Mut) to investigate its role in miR205HG function. Intriguingly, our findings indicated that ectopic miR205HG‐Mut failed to modulate the HNRNPA0 protein level (Fig. 5D,E). Altogether, our results demonstrated that LIN28A involves the mechanism of miR205HG suppressing *HNRNPA0* mRNA translation.

**Table 3:** Potential miR205HG interacting proteins identified by RNA pull‐down/MS. PSM, peptide‐spectrum match; AAs, amino acid length; GFP: spectral counts of proteins in GFP transcript group; miR205HG: spectral counts of proteins in miR205HG group; Change index = (∑(miR205HG PSM) × 100)/(∑(GFP PSM) × AAs), showing the relative enrichment of peptides in MS.

Protein	UniProt ID	AAs	GFP PSM			miR205HG PSM			Change index
			#1	#2	#3	#1	#2	#3	
TAF15	Q16514	161	1	0	2	4	7	7	3.727
LIN28A	Q9H9Z2	209	2	0	1	9	6	5	3.190
FUSIP1	O75494	262	0	1	1	3	4	3	1.908
HNRNPH2	P55795	449	0	0	1	4	0	3	1.559
GSTO1	P78417	241	1	1	1	4	3	2	1.245
SRSF1	Q07955	248	2	2	0	4	4	3	1.109
RBM4	Q9BWF3	364	0	2	0	3	2	2	0.962
YB1	P67809	324	1	1	0	1	2	3	0.926
AUF1	Q14103	355	0	2	1	3	2	4	0.845
LIN28B	Q6ZN17	250	1	2	0	2	2	1	0.667
DPM1	O60762	260	2	0	0	2	1	0	0.577
HuR	Q15717	326	0	1	2	0	3	2	0.511
SIRT1	Q96EB6	747	2	0	1	3	2	3	0.357
PCH2	Q15645	432	1	0	2	0	3	1	0.309
SRSF9	Q13242	221	1	2	0	1	1	0	0.302
SLM2	O75525	346	2	2	2	1	2	2	0.241
HNRNPH1	P31943	449	2	2	1	1	1	3	0.223
EZH2	Q15910	746	1	2	1	1	0	4	0.168
BMI1	P35226	326	1	2	1	1	0	1	0.153
ZEB1	P37275	1124	0	2	1	0	2	3	0.148
SMAD2	Q15796	467	2	0	1	0	2	0	0.143
PTB	Q9UKA9	531	0	2	1	0	2	0	0.126
LRPPRC	P42704	1394	2	0	2	1	1	2	0.072

### 3.6 MiR205HG restrains cell migration and invasion in an HNRNPA0‐dependent manner

**Fig. 6:**
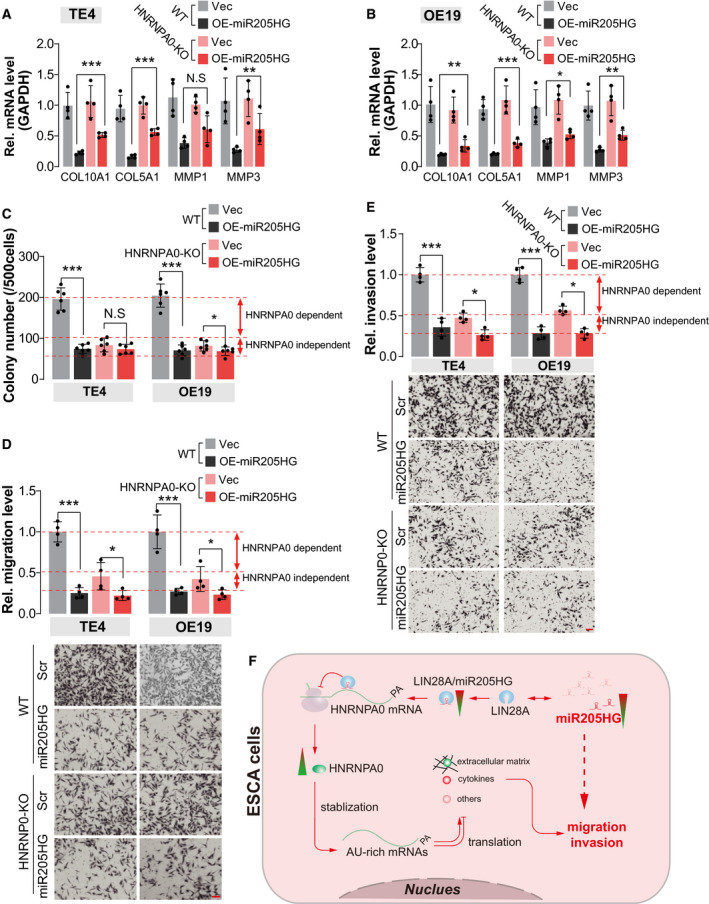
MiR205HG restrains cell migration and invasion in an HNRNPA0‐dependent manner. (A, B) mRNA level of indicated genes was determined by real‐time qPCR. Cells in Fig. S5B (HNRNPA0‐KO) were pooled together, and then overexpressed with miR205HG. Vec, vector; OE‐miR205HG, overexpression of miR205HG; *N* = 4 (mean ± SD); N.S, no significance; *P < 0.05, **P < 0.01, and ***P < 0.001 by Student's *t*‐test. GAPDH serves as the internal control. (C) Statistical results of colony formation assay using TE4 and OE19 cells. Cells in A and B were used. *N* = 4 (mean ± SD); N.S, no significance, *P < 0.05 and ***P < 0.001 by Student's *t*‐test. (D, E) Cell migration (D) and invasion (E) were determined by transwell assay. TE4 and OE19 cell lines as B and C were used. In the transwell assay of invasion, the chambers were coated using Matrigel. Scale bar, 100 µm; *N* = 4 (mean ± SD), *P < 0.05 and ***P < 0.001 by Student's *t*‐test. (F) Working model of miR205 participating in the pathogenesis of ESCA.

Considering the versatility of HNRNPA0 in influencing mRNA stability, we hypothesized that miR205HG achieves its biological functions in an HNRNP10‐dependent manner. To this end, we generated several HNRNPA0‐knockout (HNRNPA0‐KO) single‐cell clones in TE4 and OE19 cells using CRISPR/Cas9 technology (Fig. S5A,B). The HNRNPA0‐KO single clones validated by Sanger sequencing were used for further experiments (Fig. S3C). We observed that, compared with wild‐type TE4 cells, the downregulation of ECM‐related genes, such as COL10A1, COL5A1, MMP1, and MMP3, induced by miR205HG overexpression was largely blunted in HNRNPA0‐KO TE4 cells (Fig. 6A). Consistently, similar results were observed in HNRNPA0‐KO OE19 cells (Fig. 6B). These findings demonstrated that miR205HG participates in the regulation of ECM‐related gene expression in an HNRNPA0‐mediated manner, at least partially. In colony formation assay, our observation showed that the inhibitory effect of ectopic miR205HG in TE4 and OE19 cells were largely blocked (about 72.6%) by the deletion of HNRNPA0 (Fig. 6C). Moreover, in the transwell assay of migration and invasion, we found that knockout of HNRNPA0 in TE4 or OE19 cells effectively blocked (about 63.1%) the reduction of cell numbers in the bottom induced by overexpression of miR205HG (Fig. 6D,E). Therefore, our results clearly showed that HNRNPA0, at least partially, mediates the function of miR205HG in the migration and invasion of ESCA cells.

## 4 Discussion

Long noncoding RNAs, as a kind of regulatory RNA, have been reported to involve in many physiological and pathological processes in humans such as angiogenesis, tumorigenesis, and preeclampsia [32, 33, 34, 35]. Previously, the elevation of miR205HG was frequently observed in other cancer types such as lung squamous cell carcinoma, head, and neck squamous cell carcinoma, and cervical cancer [22, 36, 37]. In 2020, Li *et al*. found that miR205HG was substantially up‐regulated in (45 samples of) ESCC tissues compared with adjacent nontumor tissues [38]. In contrast, Song *et al*. recently reported that, compared with normal esophageal epithelial tissues, miR205HG is significantly downregulated in EAC [39]. In the present study, we observed that miR205HG level in ESCA tumors is downregulated compared with normal esophageal tissues, supported by three different lines of evidence: (a) recently published datasheet (GSE149609) in GEO, (b) RNAseq data of ESCA from TCGA, and (c) real‐time qPCR results of 127 ESCA tumors and adjacent normal tissues we collected. Considering the individual heterogeneity of ESCA, we definitely hold that the conclusion drawn by our study with more samples is more convincing. Also, we determined miR205HG level in nine pairs of EAC and the related adjacent nontumor tissues, and unfortunately, only a slight but not significant downregulation of miR205HG level in EAC tissues was observed. This inconsistency with the previous report may be caused by the small sample volume of EAC in our study. Altogether, we consider that the roles of miR205HG may be divergent among different cancer types.

Indeed, in previous reports, miR205HG was often proofed as an oncogene with functions such as accelerating cancer cell proliferation and promoting tumor progression [20, 36]. However, our data showed that the knockdown of miR205HG in TE6 and OE21 cells enhanced cell migration and invasion. More importantly, we observed that overexpression of miR205HG in TE4 and OE19 cells restrained ECM‐related gene expression, and knockdown of its expression in TE6 and OE21 cells exhibited opposite results. These findings clearly show the fundamental role of miR205HG in regulating ECM‐related gene expression. Previously, numerous studies demonstrated that metastasis constitutes the critical factor for cancer‐caused death of ESCA [6, 40, 41, 42, 43]. Meanwhile, metastatic behavior of cancer cells is fostered by ECM stiffness because most cells migrate faster on stiffer substrates and their persistent migration always follows the direction of stiffness gradient [44]. These lines of evidence collectively draw a scene that the downregulation of miR205HG in ESCA cells promotes the production of ECM and then enhances cancer cell migration and invasion.

Furthermore, we predicted subcellular localization and the putative targets of miR205HG to explore the underlying mechanism of how it regulates ECM‐related gene expression. iLoc‐LncRNA database predicted its subcellular location is cytoplasm or cytosol, while AnnoLnc2 predicted it localizes both in cytosol and nucleus (data not shown). These cues hint miR205HG possesses transcriptional and post‐transcriptional regulation potential. Meanwhile, our results showed that the mRNA of *HNRNPA0* may be the possible target of miR205HG, supported by at least three lines of evidence: (a) miR205HG interacts with *HNRNPA0* mRNA (RNA pull‐down assay); (b) deletion of HNRNPA0 in TE4 and OE19 cells effectively blunt the alteration in ECM‐related genes expression induced by overexpression of miR205HG; (c) miR205HG overexpression induced suppress in migration and invasion of TE4 and OE19 cells are largely blocked by deletion of HNRNPA0. HNRNPA0 encodes an mRNA‐binding component of ribonucleosomes and specifically binds ARE‐containing mRNAs [45], making it possesses the potential to regulate gene expression on a large scale (containing ECM‐related and cytokines genes). In another line, we demonstrated the miR205HG impedes the translation of HNRNPA0 but not its mRNA stability, proofed by (a) manipulation of miR205HG in ESCA cell lines merely altered HNRNPA0 protein level, and (b) HNRNPA0 protein level but not mRNA level (data not shown) were upregulated in ESCA tumors. Besides, our MS data of miR205HG RNA pull‐down products showed that miR205HG interacts with LIN28A protein, and more importantly, LIN28A participates in the regulation of miR205HG on HNRNPA0 translation. Altogether, we consider that HNRNPA0 mediates the function of miR205HG in regulating ECM‐related gene expression.

## 5 Conclusion

In summary, we first reported the expression pattern of miR205HG in ESCA tissues was downregulated when compared with normal esophageal tissues or adjacent normal tissues of tumors. Downregulation of miR205HG modulates the expression of ECM‐related genes in ESCA cells, which contributes to cell migration and invasion (Fig. 6F). In the mechanism, we demonstrated that miR205HG interacts with *HNRNPA0* mRNA, and then hamper its translation (Fig. 6F). Altogether, we highlight that miR205HG/HNRNPA0 axis is implicated in the migration and invasion of ESCA cells, which may serve as therapeutic targets to inhibit ESCA metastasis.

## Conflict of interest

The authors declare no conflict of interest.

## Author contributions

GX designed the experiments. XD, XC, DL, DD, XL, SM, and SF performed the experiments. All contributed to the writing of the paper.

## Supporting information


**Fig. S1.** MiR205HG is downregulated in ESCA.
**Fig. S2.** MiR205HG modulates ECM‐related genes expression.
**Fig. S3.** MiR205HG influences proliferation, migration, and invasion of ESCA cells.
**Fig. S4.** MiR205HG suppresses HNRNPA0 translation by interacting with LIN28A.
**Fig. S5.** miR205HG restrains cell migration and invasion in an HNRNPA0 dependent manner.
