# Supplementary material for: LncRNA miR205HG hinders HNRNPA0 translation: anti‐oncogenic effects in esophageal carcinoma
Source: Mol Oncol. 2021 Dec 20;16(3):795–812. doi: 10.1002/1878-0261.13142 (PMC8807358; doi:10.1002/1878-0261.13142)
Supplement: Supplementary file 1 — Fig. S1. MiR205HG is downregulated in ESCA. Fig. S2. MiR205HG modulates ECM‐related genes expression. Fig. S3. MiR205HG influences proliferation, migration, and invasion of ESCA cells. Fig. S4. MiR205HG suppresses HNRNPA0 translation by interacting with LIN28A. Fig. S5. miR205HG restrains cell migration and invasion in an HNRNPA0 dependent manner. [file MOL2-16-795-s001.pdf]

LncRNA miR205HG hinders HNRNPA0 translation: anti-oncogenic effects in esophageal carcinoma

Running title: Aberration of miR205HG contributes to ESCA

Supporting information

Fig. S1 MiR205HG is down-regulated in ESCA

Fig. S2 MiR205HG modulates ECM-related genes expression

Fig. S3 MiR205HG influences proliferation, migration, and invasion of ESCA cells

Fig. S4 MiR205HG suppresses HNRNPA0 translation by interacting with LIN28A

Fig. S5 miR205HG restrains cell migration and invasion in an HNRNPA0 dependent manner

Fig. S1 MiR205HG is down-regulated in ESCA

A. expression level of miR205HG in human tissues. Data were collected from Genotype-Tissue Expression Project (GTEx) database. TPM, Transcripts Per Millionin reads in RNAseq.

B. MiR205HG expression in normal esophageal tissues and ESCA tumors were analyzed by the GEPIA tool. The expression data of esophageal tissues derived from the GTEx database and the ones of ESCA tumors from TCGA were used. *p-value < 0.01.

C. MiR205HG expression of EAC biopsies was determined by real-time qPCR. 9 patients with EAC were enrolled. N.S, no significance by paired student's *t*-test.

D. Gene ontology enrichment analysis (cell component) and KEGG pathway analysis of 1181 DEGs of GSE149609.

Fig. S2 MiR205HG modulates ECM-related genes expression

A. MiR205HG expression levels in the indicated cell lines were determined by real-time qPCR. The expression level of miR205HG was normalized by GPADH (left) and ACTB (right). N=4 (mean±SD), ***p<0.001 by student's *t*-test (vs. OE21).

B-C. The indicated genes expression level in OE21(B) and FLO-1(C) cells were determined by real-time qPCR. 3 independent targets for miR205HG were used,

indicated by #1/2/3 (left). These miR205-knockdown cell lines were pooled together for the quantification of ECM-related genes (right). Src, scramble control; KD-miR205HG, knockdown of miR205HG; (B) N=6 (mean±SD), ***p<0.001 by one-way ANOVA; (C) N=3 (mean ± SD), N.S, no significance, *p<0.05 and ***p<0.001 by one-way ANOVA (left) or student's *t*-test (right).

D-E. The indicated genes expression level in OE19 (D) and SK-GT-4 (E) cells were determined by real-time qPCR. Cells were stably expressed with miR205HG (left), which then were used for the quantification of ECM-related genes (right). Vec, vector control; OE-miR205HG, overexpression of miR205HG; (left) N=6(mean ± SD), ***p<0.001 by student's *t*-test; (right) N=3 (mean±SD); *p<0.05, **p<0.01 and ***p<0.001 by student's *t*-test.

F. The protein level of the indicated proteins was determined by IB. TE6 cells are the same as Fig. 2D and OE19 cells as S2D.

Fig. S3 MiR205HG influences proliferation, migration, and invasion of ESCA cells

A-B. MiR205HG expression levels in TE6, OE21, TE4, and OE19 cells were determined by real-time qPCR. MiR205HG was stably knocked down or overexpressed. Scr, scramble control. In A, N=6 (mean±SD), ***p<0.001 by one-way ANOVA. In B, N=6 (mean±SD), ***p<0.001 by student's *t*-test.

C-D. Cell proliferation of TE4 (C) and OE19 (D) cells was determined by MTT assay. MiR205HG was stably overexpressed. N=4 (mean±SD), ***p<0.001 by two-way ANOVA.

E. Colony formation assay of TE4 and OE19 cells. Cells in A and B were used. Scale bar, 2mm; N=6 (mean±SD), ***p<0.001 by student's *t*-test.

F-G. Cell migration (F) and invasion (G) were determined by trans-well assay. Cells in A and B were used. In the trans-well assay of invasion, the chambers were coated using matrigel. Scale bar, 100μm; N=6 (mean±SD), ***p<0.001 by student's *t*-test.

Fig. S4 MiR205HG suppresses HNRNPA0 translation by interacting with LIN28A

A. The interaction between LIN28A protein and *HNRNPA0* mRNA was determined by CLIP assay. MiR205HG-knockdown TE6 and OE21 cells (1×10^7) were used. The HNRNPA0 mRNA level bound by LIN28A was determined by qPCR. Ectopic LIN2A

protein level was determined by IB (bottom). N=6 (mean ± SD), **p<0.01 and ***p<0.001 by student's *t*-test.

Fig. S5 miR205HG restrains cell migration and invasion in an HNRNPA0 dependent manner

A. Guide RNA sequences of CRISPR/Cas9 used in HNRNPA0 knockout.

B. The protein level of HNRNPA0 was determined by IB. sg, small guide RNA for CRISPR/Cas9. #1/2/3, single-cell clones.

C. Sanger sequencing validated single-cell clones in Fig. 6A. The red arrow indicated the mutation site in the *HNRNPA0* gene.

D. Colony formation assay of TE4 and OE19 cells. Cells in Fig. 6A and B were used. Scale bar, 2mm. The figure is related to statistical results in Fig. 6C.

Figure S1

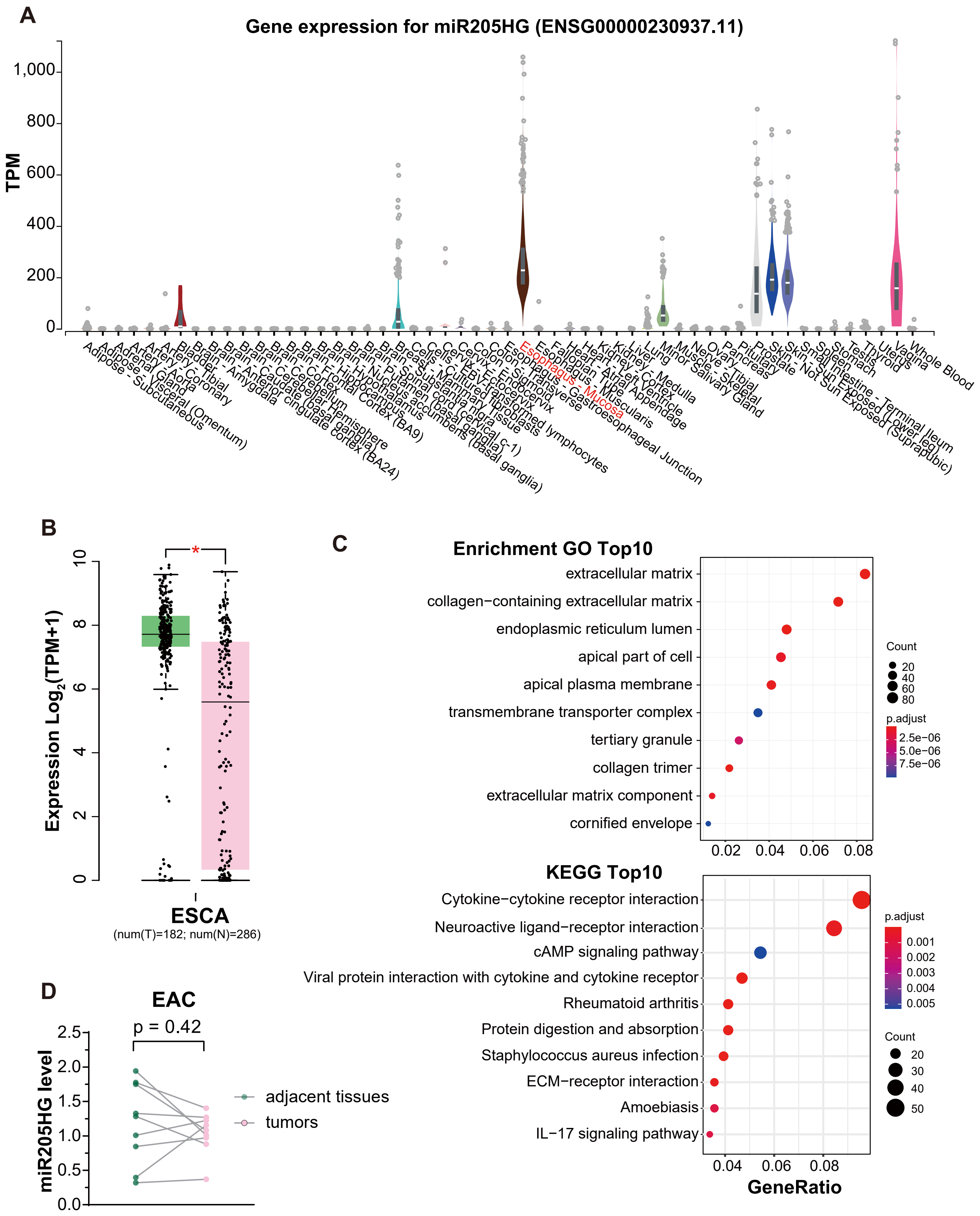

A
Gene expression for miR205HG (ENSG00000230937.11)
TPM
1,000
800
600
400
200
0
Esophagus - Mucosa
Whole Blood
B
Expression Log2(TPM+1)
*
ESCA
(num(T)=182; num(N)=286)
C
Enrichment GO Top10
extracellular matrix
collagen−containing extracellular matrix
endoplasmic reticulum lumen
apical part of cell
apical plasma membrane
transmembrane transporter complex
tertiary granule
collagen trimer
extracellular matrix component
cornified envelope
Count
20
40
60
80
p.adjust
2.5e−06
5.0e−06
7.5e−06
0.02 0.04 0.06 0.08
KEGG Top10
Cytokine−cytokine receptor interaction
Neuroactive ligand−receptor interaction
cAMP signaling pathway
Viral protein interaction with cytokine and cytokine receptor
Rheumatoid arthritis
Protein digestion and absorption
Staphylococcus aureus infection
ECM−receptor interaction
Amoebiasis
IL−17 signaling pathway
p.adjust
0.001
0.002
0.003
0.004
0.005
Count
20
30
40
50
0.04 0.06 0.08
GeneRatio
D
EAC
p = 0.42
miR205HG level
2.5
2.0
1.5
1.0
0.5
0.0
adjacent tissues
tumors

Figure S2

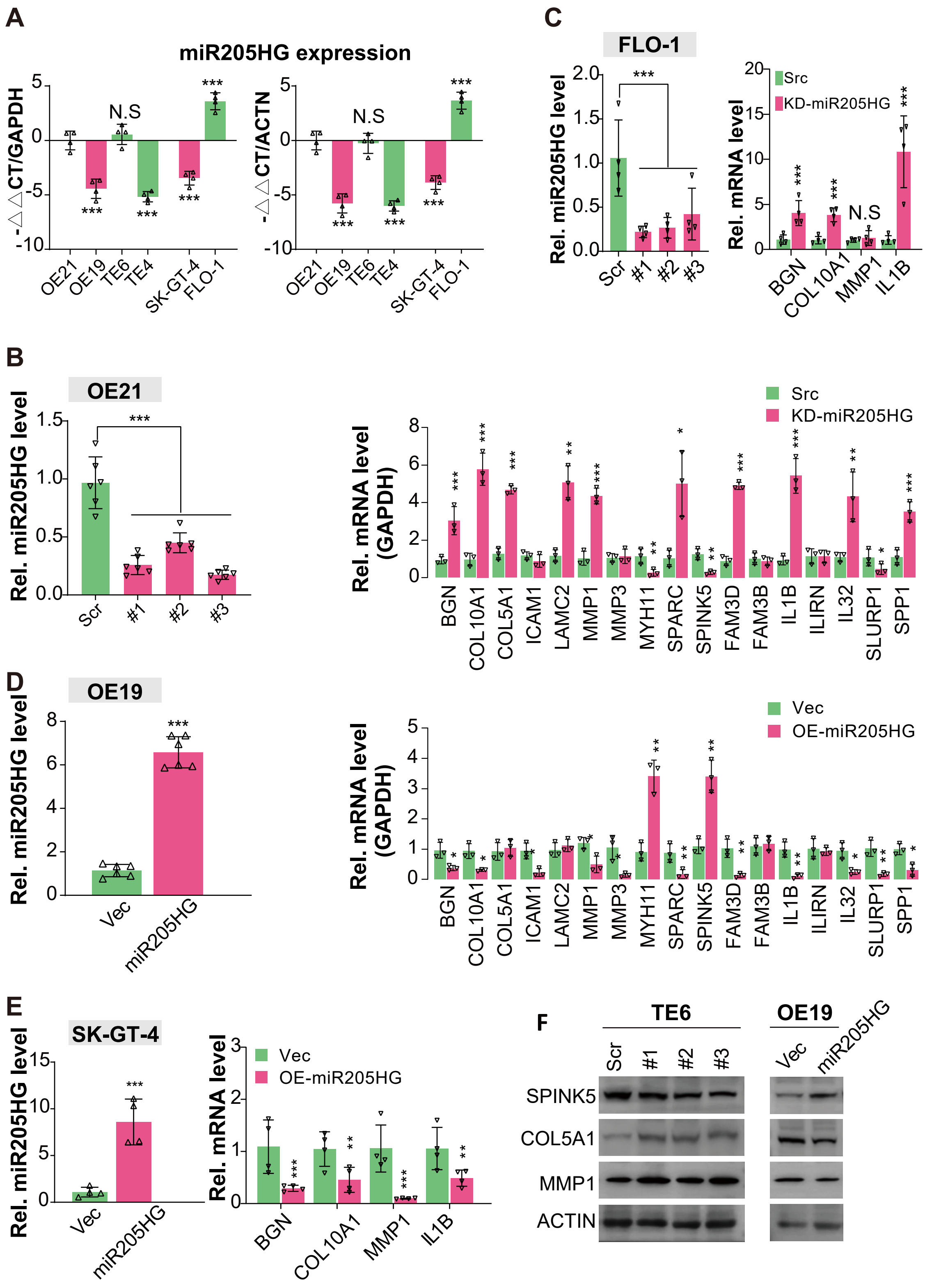
A
miR205HG expression
-△△CT/GAPDH
-△△CT/ACTN
N.S
***
OE21
OE19
TE6
TE4
SK-GT-4
FLO-1
B
OE21
Rel. miR205HG level
Scr
#1
#2
#3
Rel. mRNA level (GAPDH)
Src
KD-miR205HG
BGN
COL10A1
COL5A1
ICAM1
LAMC2
MMP1
MMP3
MYH11
SPARC
SPINK5
FAM3D
FAM3B
IL1B
ILIRN
IL32
SLURP1
SPP1
C
FLO-1
Rel. miR205HG level
Rel. mRNA level
Src
KD-miR205HG
BGN
COL10A1
MMP1
IL1B
N.S
D
OE19
Rel. miR205HG level
Vec
miR205HG
Rel. mRNA level (GAPDH)
Vec
OE-miR205HG
E
SK-GT-4
Rel. miR205HG level
Vec
miR205HG
Rel. mRNA level
Vec
OE-miR205HG
BGN
COL10A1
MMP1
IL1B
F
TE6
OE19
Scr
#1
#2
#3
Vec
miR205HG
SPINK5
COL5A1
MMP1
ACTIN

Figure S3

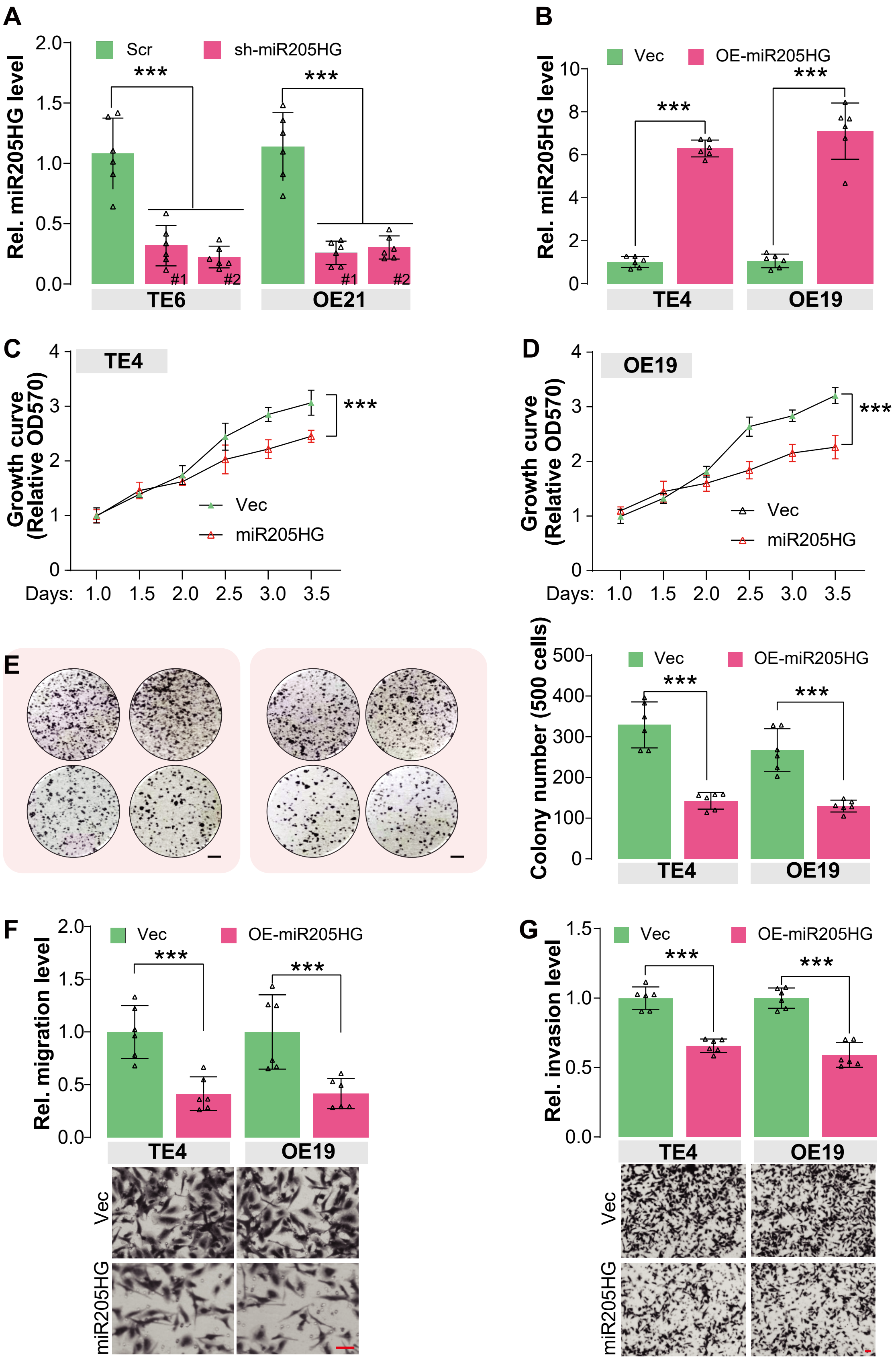
A
Scr
sh-miR205HG
Rel. miR205HG level
***
#1
#2
TE6
OE21
B
Vec
OE-miR205HG
Rel. miR205HG level
***
TE4
OE19
C
TE4
Growth curve (Relative OD570)
Vec
miR205HG
Days: 1.0 1.5 2.0 2.5 3.0 3.5
D
OE19
Growth curve (Relative OD570)
Vec
miR205HG
Days: 1.0 1.5 2.0 2.5 3.0 3.5
E
Colony number (500 cells)
Vec
OE-miR205HG
TE4
OE19
F
Rel. migration level
Vec
OE-miR205HG
TE4
OE19
Vec
miR205HG
G
Rel. invasion level
Vec
OE-miR205HG
TE4
OE19
Vec
miR205HG

Figure S4

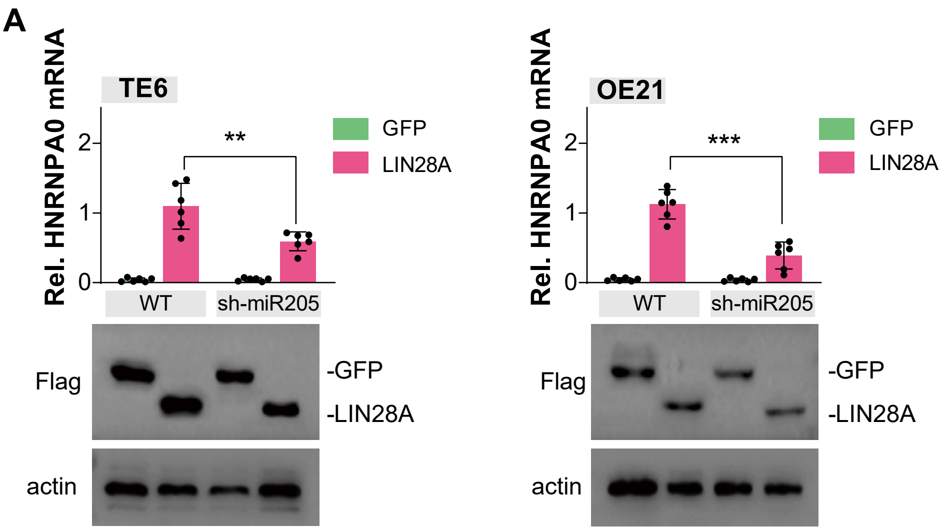

A
TE6
Rel. HNRNPA0 mRNA
2
1
0
**
GFP
LIN28A
WT
sh-miR205
Flag
-GFP
-LIN28A
actin
OE21
Rel. HNRNPA0 mRNA
2
1
0
***
GFP
LIN28A
WT
sh-miR205
Flag
-GFP
-LIN28A
actin

Figure S5

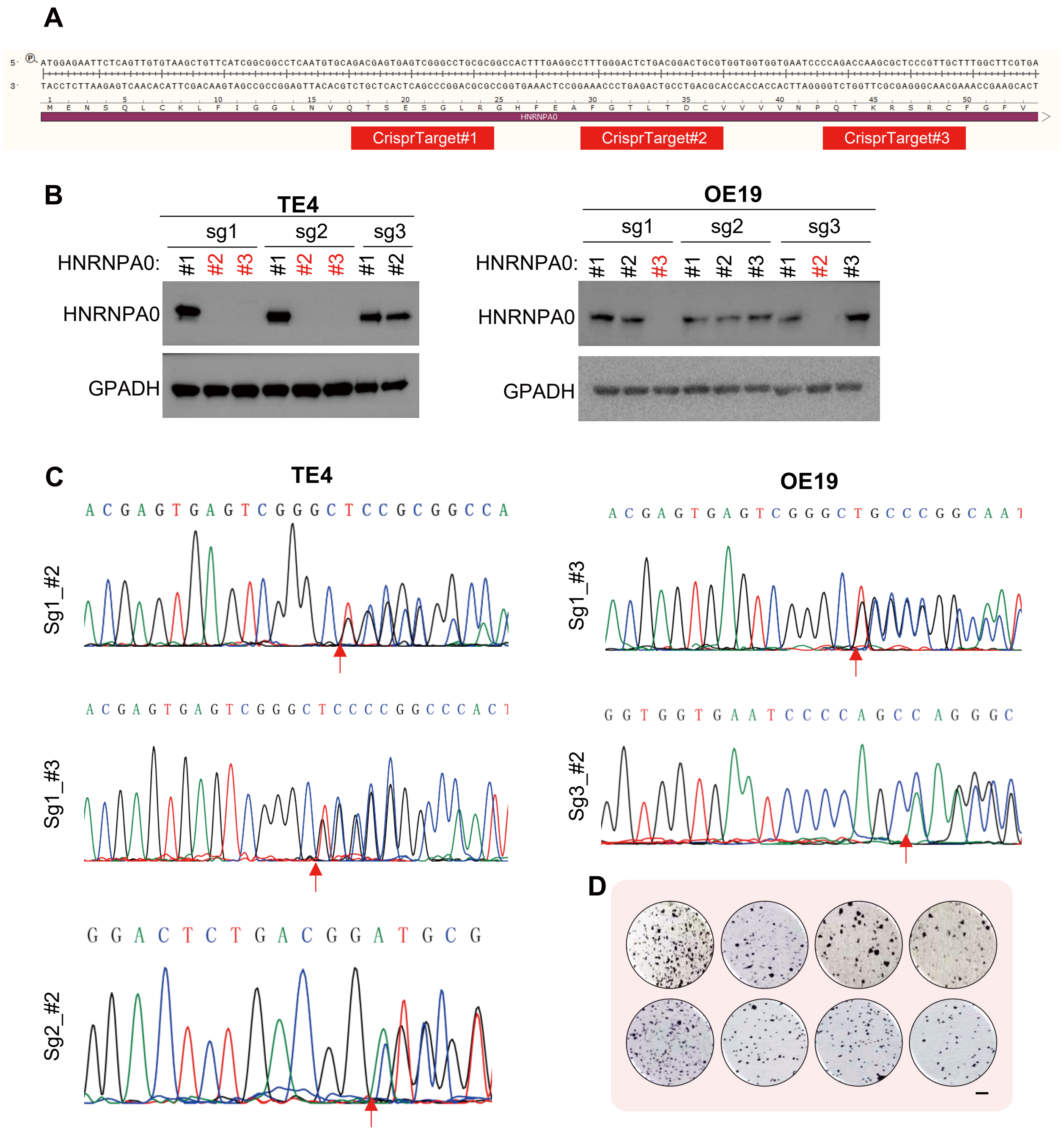
A
CrisprTarget#1
CrisprTarget#2
CrisprTarget#3
B
TE4
OE19
sg1
sg2
sg3
HNRNPA0:
#1
#2
#3
HNRNPA0
GPADH
C
TE4
OE19
Sg1_#2
Sg1_#3
Sg2_#2
Sg3_#2
D
